# In the Eye of the Beholder—Visual Search Behavior in Equestrian Dressage Judges

**DOI:** 10.3390/ani14142025

**Published:** 2024-07-09

**Authors:** Inga Wolframm, Peter Reuter, Iulia Zaharia, Johannes Vernooij

**Affiliations:** 1 Applied Research Centre, Van Hall Larenstein University of Applied Sciences, 6880 GB Velp, The Netherlands; peter.reuter@tobii.com (P.R.);; 2 Tobii Technology GmbH, 60325 Frankfurt am Main, Germany; 3 Department Population Health Sciences, Faculty of Veterinary Medicine, Utrecht University, 3584 CL Utrecht, The Netherlands; j.c.m.vernooij@uu.nl

**Keywords:** visual search behavior, dressage judging, eyetracking, equestrian sports, attention patterns

## Abstract

**Simple Summary:**

This study explored how dressage judges focus their attention on different parts of horse-rider performances during competitions. By using eye tracking technology, we analyzed where judges look and how long they focus on specific areas. We included twenty judges with varying levels of experience and recorded their eye movements as they assessed Grand Prix dressage tests on video. We found that all judges mostly looked at the front of the horse compared to the rider or other parts of the horse. However, advanced level judges paid more attention to the horse’s feet, while judges engaged at the lower level of the sport looked more at the rider. These patterns suggest that judges concentrate on a few highly relevant areas, depending on the underlying criteria for evaluating performances. Understanding judges’ visual patterns and how they interpret what they see can help improve judging, making it more accurate and transparent, ensuring more consistent evaluations in competition and improving equine welfare.

**Abstract:**

This study investigated the visual search behavior of equestrian dressage judges at different expertise levels during the assessment of Grand Prix horse-rider combinations. Twenty judges (11 foundational level, 9 advanced level) participated in the study, with their eye movements recorded using Tobii Fusion Eyetracker as they evaluated video recordings of dressage tests. Fixation metrics, namely Total Duration of Fixation (TDF), Average Duration of Fixation (ADF), and Total Number of Fixations (TNF), were analyzed across four Areas of Interest (AOIs): front, back, rider, and horse’s feet. Statistical analysis utilized linear mixed-effects models. Results demonstrated that judges consistently focused more on the front of the horse, with additional differences in fixation duration and frequency based on judge experience and specific movements. Advanced judges focused more on the horses’ feet, suggesting they draw meaning from specific areas indicative of performance quality. Conversely, foundational level judges focused more on the rider, reflecting different evaluative priorities at lower levels of the sport. These findings suggest that judges focus on a limited number of highly relevant areas, differing across movements and expertise levels. The study underscores the necessity of understanding both gaze behavior and subsequent interpretations of visual information to increase judging transparency, fairness, and equine welfare.

## 1. Introduction

The technology of eye tracking has proven to be a powerful tool for understanding human visual perception, attention, and subsequent decision-making [1,2]. Whenever people look at their surroundings, they consciously or subconsciously focus on only a limited amount of the information available. This limited focus is what is commonly referred to as “visual attention” and can be measured using fixations (i.e., a prolonged pause of the eye movement on a particular element in the visual field) and saccades (i.e., the rapid movements between fixations) [3]. Gaze behavior has been shown to generally reflect the direction of attention. It is widely assumed that it is impossible to shift the point of gaze without also shifting attention [4].

Over the years, significant strides have been made in applying eye tracking to various domains, such as psychology, neuroscience, and human-computer interaction, to study visual attention and cognitive processes, including sports performance analysis [5]. Eye tracking technology has been used as a diagnostic tool to analyze relevant visual search strategies or hand-eye coordination in professional sports [6]. Wood and Wilson [7] and Adolphe, Vickers, and Laplante [8] employed eye tracking in structured training programs, involving video feedback of gaze behavior and on-court training. Research by Savelsbergh, van Gastel, and van Kampen [9] demonstrated how gaze behavior and anticipation efforts may be improved through the acquisition of different gaze patterns. For instance, eye tracking has been used to analyze athletes’ visual strategies, such as how soccer players scan the field or how shooters focus on their targets. The technology provides objective data on where individuals look, for how long, and in what sequence, thereby offering insights into their attentional focus and information processing [9,10]. Over the years, eye tracking technology has also been used to examine the visual search strategies of sports officials and referees [11,12,13]. After all, most, if not all, judges are required to take decisions in a highly pressurized environment with a wealth of information to consider in a limited amount of time [14]. To be able to make accurate judgments, officials must be able to select and process informational cues that are most relevant to that particular situation [12,15].

Investigating the visual search behavior of officials involved at different levels of the sport is therefore thought to be a meaningful strategy for developing more effective decision-making capabilities [14,16,17,18]. Studies in gymnastics, for example, suggest that more experienced judges generate more fixations in total while also fixating comparatively more often on specific anatomical parts of the athlete, such as the head and arms [19,20,21]. In a study involving high- and low-level ice hockey referees assessing video clips, the more experienced referees were more accurate in their decision-making, yet no differences in gaze behavior could be found between the two groups [22]. The authors argue that visual assessment strategies differ when assessing sporting performances. At the same time, high-level referees may have developed more cognitive processes that allow them to extract relevant visual information more effectively (e.g., [23]). As Haider and Frensch [24] have argued in their Information Reduction hypothesis, as individuals become more skilled in a task, they process information more efficiently by focusing on relevant cues and filtering out irrelevant information. As such, it may well be the case that expert officials are able to extract critical information (visual cues) more quickly, allowing them to anticipate more effectively than novices [9,10,25].

Performance in equestrian sports depends on the harmonious yet highly intricate interaction between the horse and the rider [26]. In equestrian dressage, judges are tasked with evaluating the performance of horse-rider combinations based on a set of predefined concepts [27,28].

Judges must be able to provide accurate and relevant feedback on the quality of movements, the accuracy of execution, and the level of harmony between horse and rider, in line with the principles of ethical horse training [29]. Equestrian judges generally receive extensive training based on the principles of classical dressage, commonly referred to as the Training Scale. These six interdependent and progressive criteria aim to develop a horse’s physical and mental aptitudes, preparing it step by step for the different levels of dressage: ‘Rhythm’ refers to the consistency of each pace, maintained at a steady tempo. ‘Suppleness’ reflects the fluidity of the horse’s movements across various movements and in response to the rider’s aids. The term ‘Contact’ denotes the gentle, continuous connection between the rider’s hand and the horse’s mouth. ‘Impulsion’ describes the controlled release of energy by the horse in order to move forward. ‘Straightness’ indicates the alignment of the horse’s hindquarters with its front, on straight lines as well as when bent. ‘Collection’, as the last and most advanced step of the training scale, involves the increased engagement of the hind legs and a lowering of the hindquarters, which gives the forehand a seemingly lighter appearance. Although these criteria do not directly address horse welfare, the overarching principles of the FEI mandate that the welfare of the horse is fundamental in all equestrian training and activities [28,30,31].

When assessing horse-rider combinations in competition, judges are currently required to take all of these elements into account and assess them in relation to a set pattern of increasingly advanced movements [27,28]. However, the complexity of such judging tasks has been argued to exceed human cognitive capacity [32,33,34], resulting in the development of cognitive heuristics, commonly referred to as short-cuts, in order to cope with high cognitive load, executed under time pressure [15,35,36,37]. Some of these cognitive short cuts have been shown to result in systematic errors and biases, such as nationalistic bias [35], patriotism by proxy, home bias, reputation, and order bias [15]. While equestrian federations do produce lengthy manuals, describing each movement in great detail, there is, as yet, a lack of specific, non-arbitrary descriptions of the most salient aspects or elements of performance, including relevant welfare parameters. As a result, there is little consensus on precisely which aspects of the horse-rider combination judges should focus on. At times, this may even lead to horse-rider combinations being awarded high scores for their performances, even though certain aspects of that performance do not align with current understandings of equine welfare [38,39,40,41,42,43]. For example, a recent study by Kienapfel et al. [40] investigating horse-rider combinations competing at an international Grand Prix dressage competition showed that higher judging scores were positively correlated with nasal planes held behind the vertical. Such head-neck positions were also associated with unusual oral behavior and conflict behavior. Furthermore, results also showed that riders whose horses showed more unusual oral behavior and head-neck positions behind the vertical ranked higher in the FEI world ranking. Similarly, a study by Hamilton et al. [43] investigating conflict behaviors in horses competing in lower level dressage showed that horses with nasal planes on or behind the vertical received higher scores. While the study did not find positive correlations between higher scores and conflict behaviors, it also failed to show any negative relationships, suggesting that judges either failed to see such behavioral expressions or did not interpret them correctly. Thus, in an attempt to foster continued transparency in equestrianism and promote a continued focus on equine welfare, especially given the welfare concerns leveled at equestrianism [44,45,46,47] understanding what judges currently focus on when assessing horse-rider combinations is essential.

An earlier study by Wolframm et al. [48] provided some initial insights into the visual search strategies of Grand Prix judges. However, as yet little is known about the visual gaze behavior of dressage judges, including which elements of the horse-rider combination dressage judges focus on. Therefore, the aim of the current study was to explore visual search behavior and attentional patterns in equestrian dressage judges at different levels and across different movements, drawing on duration and frequency of fixations.

## 2. Materials and Methods

### 2.1. Participants

Twenty dressage judges, judging at foundational (N = 11) and advanced (N = 9) levels, were recruited to take part in the study. “Foundational level” judges were those qualified to assess horse-rider combinations starting out in the sport, competing at entry level to medium level. “Advanced level” judges were those qualified to assess at the higher levels, where horse-rider combinations are expected to demonstrate advanced movements. Recruitment took place on two separate days at an equestrian event. No personal information was collected, and all participants provided written consent to participate in the study.

### 2.2. Data Collection

Participating judges were asked to judge from video two Grand Prix horse-rider combinations competing at the 2023 Dutch national championships and performing the FEI GP dressage test. In order to ensure that judges assessing at any level of the sport were able to participate in the study with reasonable confidence, only the three basic paces and their extensions were included in the analysis: collected walk, extended walk, collected trot, extended trot, collected canter, and extended canter. These six movements encompass the basic paces of a horse, including extensions. Judges were asked to judge the total of 12 movements according to the rules of the Dutch equestrian federation, providing scores from 0 (=not performed) to 10 (=excellent). However, seeing that none of the judges were officially qualified to judge at the Grand Prix level and the main purpose of this study was to examine visual search strategies, their scores were not included in the advanced statistical modeling, so as not to evoke the wrong conclusions.

#### 2.2.1. Experimental Set-Up

Judges’ eye movements were recorded using the Tobii Fusion Eye tracker (at 250 Hz frequency). At the start of the experiment, the eye tracker was calibrated according to the manufacturer’s guidelines. Judges were then shown an introductory slide outlining once again the purpose of the experiment, with instructions to judge as they normally would. Having read the information, judges would then press enter and get to see another slide that indicated which movement they were about to see. Judges would then see the video snippet of the movement and say out loud the score they would give. One of the researchers would then note down the score. The subsequent 11 movements were presented in the same way, with a final slide thanking the judges for their participation.

**Figure 1:**
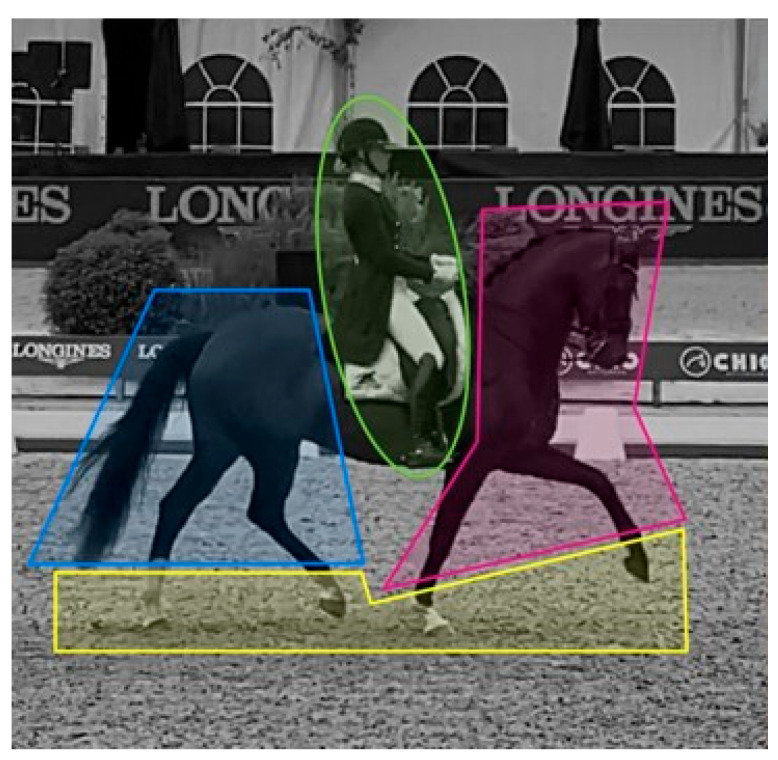
The four AOIs used for analysis: Blue = back end of the horse, Red = front of the horse, Yellow = feet of the horse, Green = rider.

For eye tracker analysis, Times of Interest (TOI) were determined for each video, including the beginning of each movement as defined in the FEI GP Dressage test. The Areas of Interest (AOIs) were drawn in the Tobii Pro Lab software, Version 1.217, outlining separate areas of the horse or rider a judge may focus on while judging. These areas were subsequently combined into four AOIs (Figure 1): front (of the horse), back end (of the horse), rider, and (horse’s) feet.

#### 2.2.2. Tobii Metrics

Tobii Pro Lab uses a filtering algorithm to process gaze data. It classifies eye movement based on the velocity of the directional shifts of the eye. The velocity is the most commonly given in visual degrees per second (°/s). When the eye movement velocity is below a certain threshold, the samples are classified as part of a fixation. For the current study, the Tobii Pro I–VT filter was set at a threshold of 30 degrees/second. The fixation filter was created to handle eye tracking data in controlled studies with minimal head movement (the Velocity-Threshold identification fixation filter), as described by Salvucci and Goldberg [3] and Komogortsev [49]. Total Number of Fixations (TNF) and Total Duration of Fixations (TDF) were recorded for each of the four AOIs. The Average Duration of Fixation (ADF) was calculated by dividing the TDF by the TNF.

### 2.3. Data Analysis

The outcome variables TNF, TDF, and ADF for each of the six movements (collected and extended walk, trot, and canter) were analyzed using a linear mixed effects model (library lme4 [50] with explanatory factors Judge Experience (JE: foundational or advanced), Area of interest (AOI: back, front, feet and rider), the interaction between both and Horse-Rider Combination (HRC) with Participant as random intercept to account for repeated observations by participant.

The validity of the models for TDF and ADF was studied for normality and homoscedasticity using libray DHARMa [51], and no aberrations were observed. For outcome TDF, a log10 transformation after addition of 100 was needed (log10(duration + 100)) to remove the zeroes. The average duration was calculated as the total duration divided by the number of fixations + 1; an addition of one to the number of fixations was added as sometimes no fixations were made. A backward selection approach by maximum likelihood was applied to obtain the most parsimonious model using Akaike’s Information Criterion (AIC). A model term was removed from the model if the AIC decreased (or maximal increased + 2) for the simpler model. AOI and judge experience were forced into the model to answer the aims of the study.

The estimates with 95% confidence intervals of the final model were estimated with REM, back transformed to the original scale for outcome TDF (antilog, 10 estimate), and presented as results. The interpretation of the back-transformed estimates is the geometric mean of TDF (intercept = reference level) and the ratio of means, i.e., when the ratio is 2 then the mean of the specified category is twice as high as the mean of the reference category. A ratio of unity (1) means equal means. The estimates of the models for ADF must be interpreted as differences between means.

The same model approach was applied for TNF, but using a generalized linear mixed model (library lme4) with a negative binomial distribution to account for overdispersion. The back transformation of the final model was performed by taking the antilog (estimate) for interpretation, which is similar to the previous description for TDF. The models were applied using R version 4.2.3 [52], and the library ggplot2 [53] was used for the visualization of the data. In order to test the validity of the statistical models, single-term deletion and likelihood ratio tests were used throughout.

## 3. Results

### 3.1. Collected Walk

#### 3.1.1. TDF

For TDF, AOI and JE were retained in the final model. Judges focused almost three times longer on the front of the horse compared to the feet, almost six times longer on the front compared to the back end, and almost 12 times longer on the rider (see Table 1).

#### 3.1.2. ADF

ADF, AOI, and JE were kept in the final model. Judges’ average fixations lasted the longest, albeit marginally so, when focused on the feet, compared to the front and the back end, yet considerably longer compared to the rider (see Table 2).

#### 3.1.3. TNF

For TNF, the factors AOI, JE, and HRC were retained in the final model. Judges focused four times more often on the front of the horse compared to the feet, six times more often than on the back end, and twelve times more often than on the rider. Judges also fixated 1.3 times more often on Horse-Rider Combination 2 (see Table 3).

### 3.2. Collected Trot

#### 3.2.1. TDF

For TDF, HRC and the interaction JE:AOI were retained in the final model. Foundational judges focused longer on the front of the horse and the rider, yet shorter on the feet compared to the back end. Advanced judges focused longer on the front and back end of the horse compared to foundational judges, yet 38 times longer on the feet and only half as long on the rider. Foundational level judges, on the other hand, focused only half as long on the back end compared to advanced judges. All judges focused comparatively longer on HRC 2 (see Table 4).

#### 3.2.2. ADF

For ADF, AOI and JE were retained in the final model. Judges’ average fixation on the front lasted the longest, followed by fixations on the back end and the rider, with considerably shorter fixations on the feet (see Table 5).

#### 3.2.3. TNF

For TNF, factors AOI, JE, and HRC were retained in the final model. Again, judges focused more often on the front, followed by the rider, the back end, and the feet. Also, judges focused more often on Horse-Rider Combination 2 (see Table 6).

### 3.3. Collected Canter

#### 3.3.1. TDF

For TDF, AOI and JE were retained in the final model. Judges focused almost seven times longer on the front compared to the feet, four and a half times longer than on the back end, and four times longer than on the front (see Table 7).

#### 3.3.2. ADF

Similarly, for ADF, AOI and JE were kept as key predictors. Average fixations on the front of the horse and the rider were longer and on the feet shorter compared to those on the back end of the horse (see Table 8).

#### 3.3.3. TNF

Lastly, for TNF, the interaction JE:AOI was retained in the model. While both foundational and advanced level judges focused considerably more often on the front of the horse compared to the other AOIs, foundational level judges focused more often on the rider and less often on the feet compared to advanced level judges (see Table 9).

### 3.4. Extended Walk

#### 3.4.1. TDF

For TDF, AOI and JE were retained in the final model. Judges focused the longest on the front of the horse, followed by the feet, yet considerably shorter on the back end and the rider. Foundational level judges had a somewhat longer total duration of fixation than advanced judges (see Table 10).

#### 3.4.2. ADF

Again, for ADF, AOI and JE were kept in the final model. The average fixation on the front of the horse lasted almost twice as long compared to the back end, followed by the feet, yet marginally longer for the rider (see Table 11).

#### 3.4.3. TNF

Lastly, for TNF, AOI and JE were retained in the final model. Judges also focused more often on the front of the horse, followed by the feet, yet considerably less often on the rider and the back end (see Table 12).

### 3.5. Extended Trot

#### 3.5.1. TDF

For TDF, AOI and JE were once again retained in the final model. Judges focused longest on the front, followed by the back end, the rider, and the feet (see Table 13).

#### 3.5.2. ADF

Equally, for ADF, AOI and JE were retained in the final model, with the average fixation lasting the longest when aimed at the back end, followed by the front, feet, and rider (see Table 14).

#### 3.5.3. TNF

Also, for TNF, AOI and JE were retained in the final model, with judges fixating twice as often on the front compared to the back end, followed by the feet and the rider (see Table 15).

### 3.6. Extended Canter

#### 3.6.1. TDF

For TDF, HRC and the interaction JE:AOI were retained in the final model. Both foundational and advanced level judges focused the longest on the front, followed by the back end, yet foundational level judges fixated six times longer on the rider compared to the feet, while advanced level judges focused one and a half times as often on the feet compared to the rider. Foundational level judges fixated the longest and most often on the front of the horse, followed by the back end, the rider, and the feet. Judges also focused considerably longer on HRC 1 (see Table 16).

#### 3.6.2. ADF

For ADF, the JE:AOI was kept in the final model. Interestingly, the average duration of fixations for foundational and advanced level judges were longest on the back end, followed by the front. Yet for foundational level judges, the average duration on the rider was three times as long as that on the feet, while advanced judges had a longer average fixation on the feet compared to the rider (see Table 17).

#### 3.6.3. TNF

Lastly, for TNF, HRC and interaction JE:AOI were retained in the final model. While once again, foundational and advanced level judges looked more often to the front, followed by the back end, foundational level judges fixated four times as often on the rider than the feet, while advanced level judges frequented the rider and the feet (almost) equally as often. Advanced judges also looked more frequently at the feet compared to foundational level judges. All judges fixated longer and more often on Horse-Rider Combination 1 (see Table 18).

## 4. Discussion

The primary aim of the study was to examine the visual search behaviors and attentional patterns of equestrian dressage judges, focusing on different movements and levels of judging expertise. Current results highlight the importance of certain anatomical areas over others and demonstrate judges’ propensity to focus on the front of the horse, irrespective of the nuanced differences across various movements.

Findings align with previous research by Wolframm et al. [48], which demonstrated that during trot and canter movements, Grand Prix judges significantly prioritized the front of the horse over the back end of the horse or the rider. These findings are particularly interesting when considered in the context of equine training. The hindquarters of the horse are considered essential and are colloquially referred to as the “engine” of movement [54,55]. By teaching a horse to lower its croup, flex its hindlegs, and place them under its center of mass while pushing off actively and at the same time working into a soft contact, it will develop ‘impulsion,’ i.e., a measure of controlled yet forward-moving energy, as well as the ability to carry itself [56,57]. This combination of self-carriage, propulsive yet contained energy, and relaxation are considered the cornerstones of good dressage and essential to achieving both collected and extended paces [27,28,54,58,59,60]. As such, it may easily be assumed that judges will tend to focus on the back end of the horse to determine whether the horse is actually lowering its hindquarters and producing the required levels of energy. However, the front of the horse, particularly how it carries its head and neck, the lift through the shoulder [54,57,58], and the quality of the contact [42,61], are likely to provide immediate and visually accessible cues of the horse’s engagement, self-carriage, and levels of relaxation [42,54,56,58].

Considering the limited time judges have to make decisions, strategic gaze patterns that rely on the most informative visual inputs while disregarding less critical data are essential [12,14]. Judges will have to prioritize those features that, to them, offer the most immediate and relevant visual information. Such an interpretation is in line with Haider and Frensch’s Information Reduction hypothesis [24] and is further supported by other research highlighting the importance for judges to engage with visual cues essential for detailed processing of relevant information [1,2,62]. As the findings from several studies examining equine behavioral parameters during dressage competitions seem to indicate [40,41,43], conflict behaviors do not currently present sufficiently salient cues to significantly impact performance scores, possibly because they are being overshadowed by the performance parameters endorsed by current criteria [27,28].

Nevertheless, current findings also provide indications for a more nuanced comparison of visual search strategies between different levels of judges. In those movements in which the horse has to actively engage its hindquarters in order to achieve the level of impulsion and degree of self-carriage required to perform the movement well, namely the collected trot, collected canter, and extended canter, differences in gaze behavior between judges at the foundational compared to the advanced level became apparent. Advanced judges focused longer and more often on the horse’s feet in, respectively, the collected trot and collected canter. In extended canter, the total and average duration, as well as the number of fixations on the feet, were all greater for advanced judges compared to judges at the foundational levels of the sport. Conversely, in these three movements, foundational level judges paid comparatively more attention to the rider.

These findings tend to align with research from Bard et al. [20] and Pizzera et al. [19], who investigated gaze patterns in gymnastic judging. The authors found that experienced judges focused more on the gymnast’s head and arms, while novice judges paid more attention to the gymnast’s legs. The implication here is that, at an advanced level, judges are able to draw meaning from very specific areas of interest because they provide a more nuanced and subtle indication of the quality of the entire performance. Following such a line of argument, current findings would suggest that looking at, for instance, the horse’s feet is a more effective approach when assessing horse-rider performance. The Federation Equestre Internationale describes collection through the shortening of the strides with the same levels of activity, resulting in a more cadenced appearance of the gait [27]. In the extension, the horse is required to lengthen its frame and cover more ground without the steps becoming hurried. It could therefore be argued that both for the collected and extended gaits, the feet provide essential clues on how to assess the quality of the movements. As has been argued by Spitz et al. [63], highly advanced officials develop more elaborate knowledge structures, allowing them to extract more detailed information from very specific aspects of athletic performances and be able to predict how subsequent performance might unfold [63,64]. Advanced dressage judges could be argued to have developed more refined assessment strategies to judge horse-rider combinations. It should be borne in mind, though, that these knowledge structures will have developed over the past few years and be primarily based on the judging principles established by the FEI and national governing bodies [28,65]. Behavioral parameters indicative of a horse’s welfare state rather than performance, such as head-neck positions [39,40,42], excessive oral behavior [40], or tail swishing [38,40], do not, at present, feature in those judging guidelines. It stands to reason, therefore, that welfare parameters are not (yet) considered sufficiently pertinent to affect gaze behavior or subsequent judging decisions.

In equestrian sports, the level of horse-rider performance depends on two athletes, the rider and the horse. At the lower levels of the sport, the rider or the horse (or both) are merely at the beginning of their journey to develop the necessary physical (and mental) aptitude to perform in dressage. Horse-rider combinations performing at the higher levels of the sport are required to demonstrate a more sophisticated level of training. It might therefore be argued that differences in gaze behavior between advanced and foundational level judges are due to the task-specific demands required of them when judging at their usual level. At an advanced level, judges are used to seeing and assessing horse-rider combinations capable of showing degrees of collection. As a result, judges may focus more on the details that are indicative of the horse’s ability to perform those higher-level movements, such as the placement of the horse’s feet, in order to gauge the quality of collection and extensions. At the foundational level, however, judges need to pay more attention to the rider, as, at that level, riders are still developing either their own skill set or that of their horse. As such, the way they interact with their horse through their seat, legs, and hands becomes more important to evaluate. Differences in gaze behavior might therefore not merely be a reflection of a lack of proficiency but might, in fact, be indicative of different evaluation criteria for advanced compared to foundational levels. Findings from Kienapfel et al. [42] might provide additional evidence. The authors demonstrated that a head-neck position behind the vertical was penalized at lower levels of dressage but not at higher levels. As findings from the current study demonstrated, lower level judges tend to focus more on the rider. As a result, they might be more prone to seeing the rider using inappropriate, heavy-handed aids that effectively pull the horse’s head behind the vertical. The lower scores given by the lower level judges in Kienapfel’s [42] study might therefore be in reference to poorer riding skills, while at the same time penalizing inappropriate head-neck positions. Advanced judges, on the other hand, tend to focus significantly less on the rider, paying more attention to cues that are primarily performance-oriented, rather than indicative of welfare.

Interestingly though, current findings show no significant difference in how advanced and foundational level judges assess the walk. On average, judges focused twice as often on the front of the horse, with the average duration of fixations being as long on the front as on the feet. The rider and the back end of the horse received comparatively little attention. The walk is a gait without a suspension phase, characterized by four separate, equally spaced hoof placements. The horse must also be fully relaxed, accepting the bit without hesitation or resistance. As the walk has no suspension phase, generating the propulsive energy that can subsequently be contained and guided through a soft contact is not possible. In fact, too much or untimely interference from the rider, through the hands, legs, or an unbalanced seat, is likely to disturb the precarious rhythm of the walk and the soft, steady contact with the hand. Not surprisingly, therefore, the walk is generally considered the most challenging gait to ride well [66,67]. Judges’ fixation patterns on the front of the horse, followed by the feet, seem to indicate that these anatomical areas do indeed provide the most salient cues for judges to assess performances, regardless of level of experience [19,20,63]. By assessing the front of the horse, judges are able to assess aspects relating to relaxation and acceptance of the contact, while fixations on the feet allow judges to determine aspects of the regularity of the walk.

Lastly, the effect of Horse-Rider Combination was less consistent across gaits. Judges focused more on Horse-Rider Combination 2 in Collected Trot and Horse-Rider Combination 1 in Extended Canter, indicating variability in visual strategies based on perceived performance or characteristics of the combinations. Additional research is required to gain a more thorough understanding of which specific elements in performance cause more or less intensive gaze patterns.

Taken together, current findings provide a first indication of which areas of the horse-rider combination provide judges with the most relevant information when assessing dressage performance. What is more, the current study also demonstrates that judges do, in fact, focus on a limited number of highly relevant areas of interest. However, these areas of interest tend to differ for certain movements and between judges at different levels of the sport. Recent studies highlight the necessity of not only focusing on gaze behavior but also on interpreting the visual information gathered [68]. This dual focus has been identified as the “missing link” in elevating the effectiveness of judging [14]. particularly relevant in the context of dressage. Given that undesirable head-neck positions and expressions of conflict behavior in dressage horses have been shown to be inadequately reflected in the dressage scores given [39,40,41,42,43] it seems more important than ever to determine how judges interpret what they see. Future research should explore how assessment strategies of judges could be enhanced by opening up the discussion on precisely which aspects of the equine training scale and relevant welfare parameters, judges are assessing when focusing on specific parts of horses and riders. Providing immediate visual feedback and allowing judges to subsequently elucidate their decision-making processes might help to clarify what is meant by different training principles and how to assess them accurately, even when under time constraints. Such developments might also go some way towards lowering the cognitive load of the judging task and mitigating some of the more pronounced biases seen in dressage judging [15,35,36]. Expanding this research to include other equestrian disciplines might also offer broader insights into the application of eye-tracking technology in sports performance analysis.

While this study provides considerable insights into the visual strategies of dressage judges, it is important to note the context in which these findings were generated. The number of participants, although sufficient to detect significant patterns in gaze behavior, was limited. While mitigated by the richness of the data collected from each participant, future studies should aim to include more participants, ideally also at the highest level of the sport, in order to be able to compare visual search patterns effectively.

As the judges involved in this study were not currently qualified to judge at the Grand Prix level, it was decided not to include the scores given in the analysis. In the future, combining dressage scores with visual search behavior would provide additional insights into how gaze patterns are related to perceptions of quality.

Lastly, the current study used a screen-based system to assess visual search behavior. While such a system makes it possible to accurately compare gaze behavior between individuals, it may not always replicate exactly the visual search pattern as it would in real life [5]. Future studies should therefore focus on including dynamic eye tracking systems, which allow for the recording of visual attention in real-life settings.

## 5. Conclusions

This study examined the visual search behaviors and attentional patterns of equestrian dressage judges, focusing on various movements and levels of judging expertise. Results show that when under operational constraints, judges tend to prioritize, focusing on a limited number of highly relevant areas of interest. However, these areas of interest tend to differ for certain movements and between judges at different levels of the sport.

While the different visual search patterns of advanced and foundational level judges may be indicative of the greater expertise of advanced judges, they may also reflect differences in task-specific patterns and their subsequent interpretation by judges.

The study underscores the importance of investigating visual gaze patterns in equestrian dressage in particular and highlights the potential of eye-tracking to enhance judging accuracy and transparency, especially with a view to prioritizing welfare parameters. The findings suggest that current judging practices could benefit from the development of new guidelines incorporating these insights. By refining these guidelines, the judging process could become more transparent, helping to promote equine welfare by ensuring that horses and relevant behavioral expressions are judged more accurately and fairly.

## Figures and Tables

**Figure 2:**
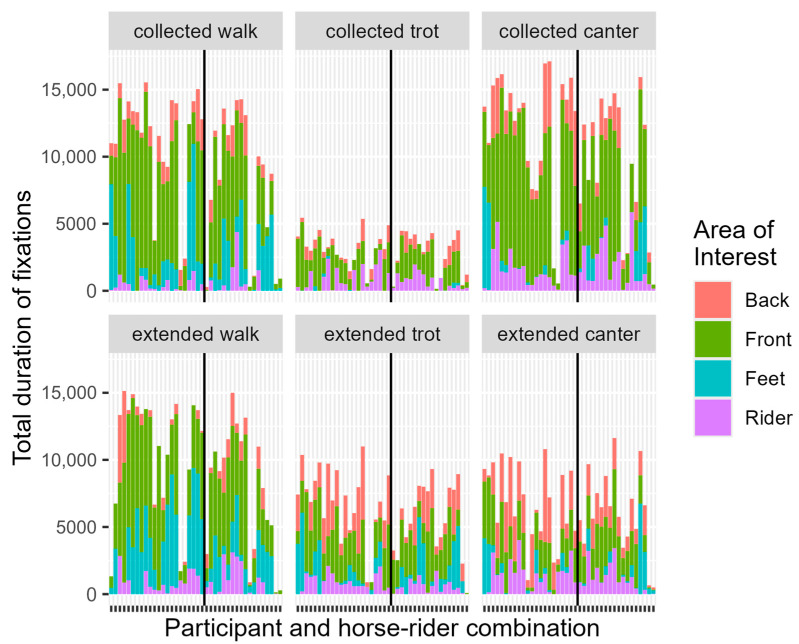
Stacked graphs of Total Duration of Fixations on Area of Interest per Participant and horse-rider combination separated per movement. The vertical line separates the horse-rider combination (left = HRC 1; right = HRC 2).

**Figure 3:**
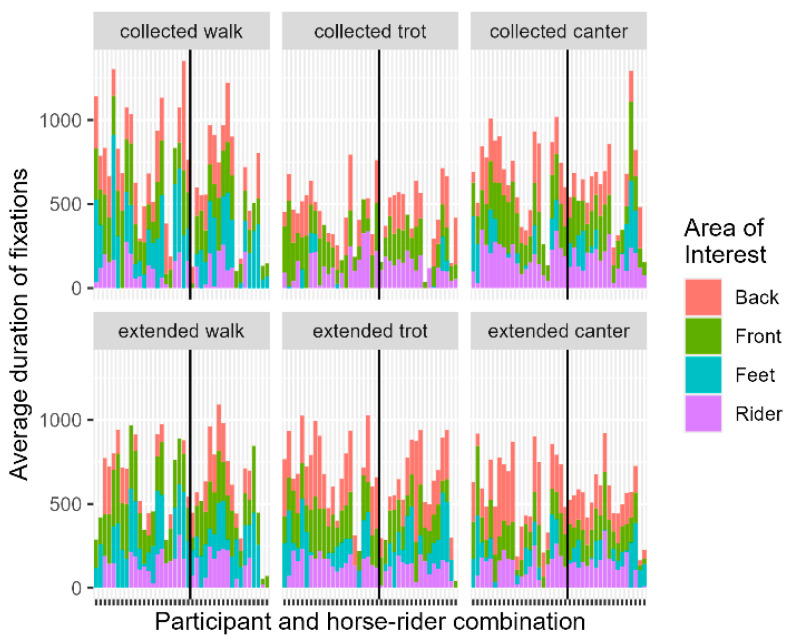
Stacked graphs of Average Duration of Fixations on Area of Interest per Participant and horse-rider combination separated per movement. The vertical line separates the horse-rider combination (left = HRC 1; right = HRC 2).

**Figure 4:**
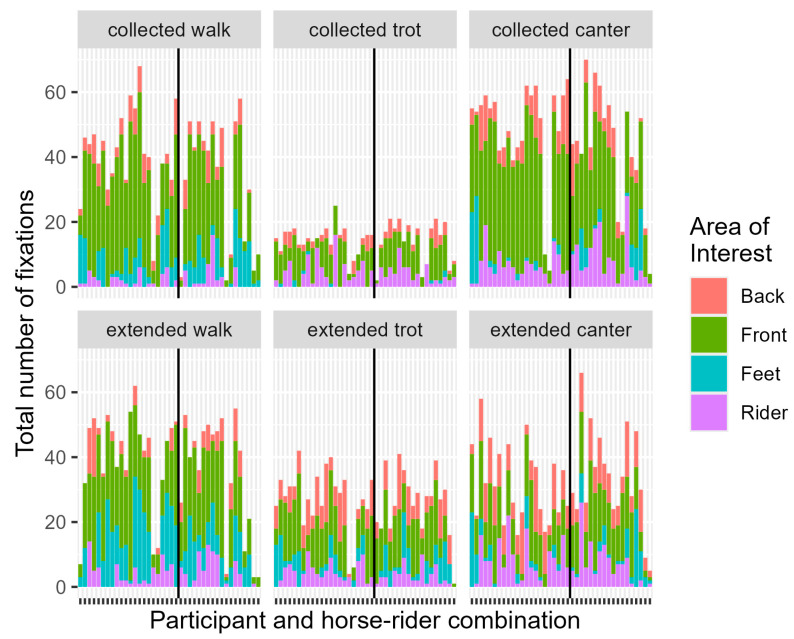
Stacked graphs of Total Number of Fixations per Area of Interest per Participant and horse-rider combination separated per movement. The vertical line separates the horse-rider combination (left = HRC 1; right = HRC 2).

**Table 1:** To be read with Figure 2—Collected Walk. Summary statistics for total duration (milliseconds) of fixation in Collected Walk and estimated ratio ^2^ between means with 95% confidence interval of the final model ^1^ per Judging Experience (JE) and Area of Interest (AOI).

Total Duration of Fixations				RATIO		
	Average	SD	Median	Ave|Ratio	2.50%	97.50%
(Mean in Ref)				786.7	460.9	1342.7
Area of Interest						
Back	1117.4	930.4	1005.0	Ref		
Front	5975.2	3839.0	5856.5	5.1	3.3	8.0
Feet	2282.6	2608.0	1308.0	1.2	0.8	1.9
Rider	465.1	800.5	133.5	0.4	0.2	0.6
Judging experience						
Foundational	2486.9	3303.1	933.5	Ref		
Advanced	2427.3	3086.1	1158.5	1.1	0.5	2.1

Ref = reference category. ^1^ Fixed effects in the full model: Judging Experience (JE) + Area of Interest (AOI) + Horse-Rider Combination (HRC) + Interaction (JE:AOI). ^2^ Ratio between mean in specified category and mean in reference category; Ratio = 1 means equal means in both categories.

**Table 2:** To be read with Figure 3 (Collected Walk). Summary statistics for average duration (milliseconds) of fixation in Collected Walk with estimated difference between means and a 95% confidence interval of the final model ^1^ per Judging Experience (JE) and Area of Interest (AOI).

Average Duration of Fixation (Duration/(Number + 1))						
	Average	SD	Median	Ave|Dif	2.50%	97.50%
(Mean in Ref)				188.6	133.7	243.4
Area of Interest						
Back	184.7	124.5	196.8	Ref		
Front	217.4	98.4	216.2	32.8	−15.8	81.3
Feet	217.7	183.1	211.5	33.1	−15.5	81.6
Rider	86.1	90.2	60.9	−98.6	−147.1	−50.1
Judging experience						
Foundational	180.4	142.0	193.5	Ref		
Advanced	171.7	136.1	159.7	−8.7	−78.5	61.2

Ref = reference category. ^1^ Fixed effects in the full model: Judging Experience (JE) + Area of Interest (AOI) + Horse-Rider Combination (HRC) + Interaction (JE:AOI).

**Table 3:** To be read with Figure 4 (Collected Walk). Summary statistics for the number of fixations in Collected Walk and estimated ratio ^2^ between means with a 95% confidence interval of the final model ^1^ per Judging Experience (JE), Horse-Rider Combination (HRC) and Area of Interest (AOI).

Number of Fixations				RATIO		
	Average	SD	Median	Ave|Ratio	2.50%	97.50%
(Mean in Ref)				3.2	2.0	5.0
Area of Interest						
Back	4.4	3.4	4.0	Ref		
Front	24.2	12.8	25.5	5.6	4.0	7.9
Feet	6.5	5.9	5.5	1.5	1.0	2.2
Rider	2.1	3.1	1.0	0.5	0.3	0.7
Horse-Rider Combination						
HRC 1	8.0	10.1	4.0	Ref		
HRC 2	10.5	12.6	5.0	1.3	1.0	1.7
Judging experience						
Foundational	8.7	10.9	4.0	Ref		
Advanced	9.9	12.1	6.0	1.1	0.6	1.9

Ref = reference category. ^1^ Fixed effects in the full model: Judging Experience (JE) + Area of Interest (AOI) + Horse-Rider Combination (HRC) + Interaction (JE:AOI). ^2^ Ratio between the mean number in the specified category and the mean number in the reference category; Ratio = 1 means equal means in both categories.

**Table 4:** To be read with Figure 2 (Collected Trot). Summary statistics for total duration (milliseconds) of fixation in Collected Trot and estimated ratio ^2^ between means with 95% confidence interval of the final model ^1^ for interaction of Judging Experience (JE) with Area of Interest (AOI) and Horse-Rider Combination (HRC).

Total Duration of Fixations (log10(Duration + 100))				RATIO		
	Average	SD	Median	Ave|Ratio	2.50%	97.50%
(Mean in Ref)				283.4	191.5	419.2
Area of Interest						
Back in Foundational	377.9	415.1	350	Ref		
Front in Foundational	1553.69	1249.6	1205.5	3.4	2.1	5.4
Feet in Foundational	35.5	93.7	0	0.4	0.2	0.6
Rider in Foundational	1004.2	835.8	946.5	2.3	1.4	3.6
Advanced compared to Foundational (Ref)						
Back in Advanced	687.8	511.8	616	1.9	1.1	3.3
Front in Advanced	1928.4	1196.2	1734.5	1.5	0.9	2.7
Feet in Advanced	1322.1	1818.7	397.5	1	0.6	1.8
Rider in Advanced	511.2	622.8	262	0.5	0.3	0.9
Horse-Rider Combination						
HRC 1	686.4	964.6	258.5	Ref		
HRC 2	842.8	990	484.5	1.3	1.0	1.7

Ref = reference category. ^1^ Fixed effects in the full model: Judging Experience (JE) + Area of Interest (AOI) + Horse-Rider Combination (HRC) + Interaction (JE:AOI). ^2^ Ratio between the mean in the specified category and the mean in the reference category; Ratio = 1 means equal means in both categories.

**Table 5:** To be read with Figure 3 (Collected Trot). Summary statistics for average duration (ms) of fixation in Collected Trot with estimated difference between means and 95% confidence interval of the final model ^1^ per Judging Experience (JE) and Area of Interest (AOI).

Average Duration of Fixation (Duration/(Number + 1))						
	Average	SD	Median	Ave|Dif	2.50%	97.50%
(Mean in Ref)				131.2	99.5	162.9
Area of Interest						
Back	132.4	97.6	142.1	Ref		
Front	175.6	93.4	189.7	43.2	8.7	77.6
Feet	16	43	0	−116.4	−150.8	−81.9
Rider	117.9	89.7	112.6	−14.5	−48.9	19.9
Judging Experience Foundational	109.3	98.9	111.3	Ref		
Judging Experience Advanced	111.9	105.8	91.6	2.6	−33.6	38.8

Ref = reference category. ^1^ Fixed effects in the full model: Judging Experience (JE) + Area of Interest (AOI) + Horse-Rider Combination (HRC) + Interaction (JE:AOI).

**Table 6:** To be read with Figure 4 (Collected Trot). Summary statistics for the number of fixations in Collected Trot and estimated ratio ^2^ between means with a 95% confidence interval of the final model ^1^ per Judging Experience (JE) and Area of Interest (AOI).

Number of Fixations				RATIO		
	Average	SD	Median	Ave|Ratio	2.50%	97.50%
(Mean in Ref)				1.6	1.2	2.4
Areas of Interest						
Back	2.2	2	2	Ref		
Front	7.8	4.1	8.5	3.7	2.6	5.3
Feet	0.3	0.5	0	0.1	0.1	0.2
Rider	4.2	3.8	3	1.9	1.3	2.7
Judging Experience Foundational	3.2	4.1	2	Ref		
Judging Experience Advanced	4	4	3	1.2	0.9	1.6

Ref = reference category. ^1^ Fixed effects in the full model: Judging Experience (JE) + Area of Interest (AOI) + Horse-Rider Combination (HRC) + Interaction (JE:AOI). ^2^ Ratio between the mean number in the specified category and the mean number in the reference category; Ratio = 1 means equal means in both categories.

**Table 7:** To be read with Figure 2 (Collected Canter). Summary statistics for total duration (ms) of fixation in Collected Canter and estimated ratio ^2^ between means with a 95% confidence interval of the final model ^1^ per Judging Experience (JE) and Area of Interest (AOI).

Total Duration of Fixations				RATIO		
	Average	SD	Median	Ave|Ratio	2.50%	97.50%
(Mean in Ref)				926.95	580.367	1480.505
Area of Interest						
Back	1508.2	1540.6	1021	Ref		
Front	6930.3	3544.1	7510	5.9	3.9	9.2
Feet	859.4	1838.2	0	0.3	0.2	0.5
Rider	1711.1	1503.9	1214.5	1.3	0.9	2
Judging Experience						
Foundational	2714.3	3464.7	1372.5	Ref		
Advanced	2798.6	3150.2	1158	1	0.6	1.9

Ref = reference category. ^1^ Fixed effects in the full model: Judging Experience (JE) + Area of Interest (AOI) + Horse-Rider Combination (HRC) + Interaction (JE:AOI). ^2^ Ratio between the mean in the specified category and the mean in the reference category; Ratio = 1 means equal means in both categories.

**Table 8:** To be read with Figure 3 (Collected Canter). Summary statistics for average duration (milliseconds) of fixation in Collected Canter with a 95% confidence interval of the final model ^1^ per Judging Experience (JE) and Area of Interest (AOI).

Average Duration of Fixation (Duration/(Number + 1))						
	Average	SD	Median	Ave|Dif	2.50%	97.50%
(Mean in Ref)				159.4	118.3	200.5
Area of Interest						
Back	158.7	97.6	154.3			
Front	220.4	83.4	209.4	61.7	26.5	97.0
Feet	81.6	114.6	0.0	−77.0	−112.3	−41.8
Rider	179.2	81.8	194.6	20.5	−14.7	55.7
Judging Experience						
Foundational	160.7	103.8	178.2	Ref		
Advanced	159.1	111.7	156.5	−1.6	−54.6	51.3

Ref = reference category. ^1^ Fixed effects in the full model: Judging Experience (JE) + Area of Interest (AOI) + Horse-Rider Combination (HRC) + Interaction (JE:AOI).

**Table 9:** To be read with Figure 4 (Collected Canter). Summary statistics for the number of fixations in the Collected Canter and estimated ratio ^2^ between means with a 95% confidence interval of the final model ^1^ per Area of Interest (AOI) and interaction between Judging Experience (JE) and Area of Interest (AOI).

Number of Fixations				RATIO		
	Average	SD	Median	Ave|Ratio	2.50%	97.50%
(Mean in Ref)				5.2	3.3	8.1
Area of Interest						
Back	5.8	4.4	5.5			
Front	28.6	12.5	29.5	5.1	3.2	8.1
Feet	1.6	3	0	0.3	0.1	0.5
Rider	8.2	5	7	1.5	0.9	2.3
Advanced compared to Foundational (Ref)						
Back in Advanced	7.5	6.6	6	1.3	0.7	2.6
Front in Advanced	29.6	11.1	30.5	1.1	0.6	2.0
Feet in Advanced	5.1	8.6	0.5	3.2	1.5	6.9
Rider in Advanced	6.4	6.8	5	0.8	0.4	1.5

Ref = reference category. ^1^ Fixed effects in the full model: Judging Experience (JE) + Area of Interest (AOI) + Horse-Rider Combination (HRC) + Interaction (JE:AOI). ^2^ Ratio between the mean number in the specified category and the mean number in the reference category; Ratio = 1 means equal means in both categories.

**Table 10:** To be read with Figure 2 (Extended Walk). Summary statistics for total duration (milliseconds) of fixation in Extended Walk and estimated ratio ^2^ between means with a 95% confidence interval of the final model ^1^ per Judging Experience (JE) and Area of Interest (AOI).

Total Duration of Fixations				RATIO		
	Average	SD	Median	Ave|Ratio	2.50%	97.50%
(Mean in Ref)				516.8	304.6	876.8
Area of Interest						
Back	802.2	1217.0	398.0			
Front	4983.8	3135.4	4636.5	8.3	5.2	13.3
Feet	2801.1	2411.4	2797.0	3.4	2.2	5.5
Rider	817.7	953.2	538.5	1.1	0.7	1.7
Judging Experience						
Foundational	2515.5	2820.3	1206.0	Ref		
Advanced	2150.3	2604.1	875.5	0.7	0.4	1.4

Ref = reference category. ^1^ Fixed effects in the full model: Judging Experience (JE) + Area of Interest (AOI) + Horse-Rider Combination (HRC) + Interaction (JE:AOI). ^2^ Ratio between the mean in the specified category and the mean in the reference category; Ratio = 1 means equal means in both categories.

**Table 11:** To be read with Figure 3 (Extended Walk). Summary statistics for average duration (milliseconds) of fixation in Extended Walk with estimated difference between means and a 95% confidence interval of the final model ^1^ per Judging Experience (JE) and Area of Interest (AOI).

Average Duration of Fixation (Duration/(Number + 1))						
	Average	SD	Median	Ave|Dif	2.50%	97.50%
(Mean in Ref)				125.2	82.5	168.0
Area of Interest						
Back	109.0	100.4	90.2			
Front	226.4	92.3	218.6	117.5	78.3	156.6
Feet	194.4	126.4	198.8	85.4	46.2	124.6
Rider	111.5	94.1	133.5	2.5	−36.6	41.7
Judging Experience						
Foundational	176.6	110.7	184.2	Ref		
Advanced	140.4	118.5	134.5	−36.1	−89.8	17.6

Ref = reference category. ^1^ Fixed effects in the full model: Judging Experience (JE) + Area of Interest (AOI) + Horse-Rider Combination (HRC) + Interaction (JE:AOI).

**Table 12:** To be read with Figure 4 (Extended Walk). Summary statistics for the number of fixations in Extended Walk and estimated ratio ^2^ between means with a 95% confidence interval of the final model ^1^ per Judging Experience (JE) and Area of Interest (AOI).

Number of Fixations				RATIO		
	Average	SD	Median	Ave|Ratio	2.50%	97.50%
(Mean in Ref)				3.2	2.0	5.1
Area of Interest						
Back	3.6	4.2	2.0			
Front	19.6	10.8	19.5	6.1	4.1	9.1
Feet	10.7	8.4	10.0	3.3	2.2	5.0
Rider	3.9	4.1	2.0	1.1	0.7	1.7
Judging Experience						
Foundational	9.6	9.5	6.5	Ref		
Advanced	9.2	10.3	6.0	0.8	0.5	1.4

Ref = reference category. ^1^ Fixed effects in full model: Judging Experience (JE) + Area of Interest (AOI) + Horse-Rider Combination (HRC) + Interaction (JE:AOI). ^2^ Ratio between the mean number in the specified category and the mean number in the reference category; Ratio = 1 means equal means in both categories.

**Table 13:** To be read with Figure 2 (Extended Trot). Summary statistics for total duration (milliseconds) of fixation in Extended Trot and estimated ratio ^2^ between means with a 95% confidence interval of the final model ^1^ per Judging Experience (JE) and Area of Interest (AOI).

Total Duration of Fixations				RATIO		
	Average	SD	Median	Ave|Ratio	2.50%	97.50%
(Mean in Ref)				1228.4	790.0	1910.1
Area of Interest						
Back	1814.5	1454.6	1422.0			
Front	2654.8	1537.0	2539.0	1.6	1.1	2.5
Feet	1124.8	1553.2	321.0	0.4	0.3	0.6
Rider	731.4	590.1	606.0	0.5	0.3	0.7
Judging Experience						
Foundational	1512.0	1416.7	1201.5			
Advanced	1666.7	1645.6	1016.0	1.1	0.6	1.9

Ref = reference category. ^1^ Fixed effects in the full model: Judging Experience (JE) + Area of Interest (AOI) + Horse-Rider Combination (HRC) + Interaction (JE:AOI). ^2^ Ratio between the mean number in the specified category and the mean number in the reference category; Ratio = 1 means equal means in both categories.

**Table 14:** To be read with Figure 3 (Extended Trot). Summary statistics for average duration (milliseconds) of fixation in Extended Trot with estimated difference between means and a 95% confidence interval of the final model ^1^ per Judging Experience (JE) and Area of Interest (AOI).

Average Duration of Fixation (Duration/(Number + 1))						
	Average	SD	Median	Ave|Dif	2.50%	97.50%
(Mean in Ref)					174.0	258.8
Area of Interest						
Back	214.1	133.1	205.7			
Front	187.0	79.1	193.9	−27.0	−68.2	14.1
Feet	132.8	124.3	115.4	−81.3	−122.4	−40.1
Rider	126.3	68.8	143.8	−87.8	−128.9	−46.6
Judging Experience (JE)						
Foundational	167.4	118.4	159.0			
Advanced	162.2	100.6	157.5	−5.2	−57.0	46.6

Ref = reference category. ^1^ Fixed effects in full model: Judging Experience (JE) + Area of Interest (AOI) + Horse-Rider Combination (HRC) + Interaction (JE:AOI).

**Table 15:** To be read with Figure 4 (Extended Trot). Summary statistics for the number of fixations in Extended Trot and estimated ratio ^2^ between means with a 95% confidence interval of the final model ^1^ per Judging Experience (JE) and Area of Interest (AOI).

Number of Fixations				RATIO		
	Average	SD	Median	Ave|Ratio	2.50%	97.50%
(Mean in Ref)				6.2	4.6	8.4
Area of Interest						
Back	6.7	4.6	6.0			
Front	12.2	6.1	13.0	1.8	1.3	2.5
Feet	4.2	4.3	2.0	0.6	0.4	0.9
Rider	3.9	3.1	3.0	0.6	0.4	0.8
Judging Experience (JE)						
Foundational	6.4	5.6	5.0			
Advanced	7.1	5.8	6.0	1.1	0.8	1.5

Ref = reference category. ^1^ Fixed effects in the full model: Judging Experience (JE) + Area of Interest (AOI) + Horse-Rider Combination (HRC) + Interaction (JE:AOI). ^2^ Ratio between the mean number in the specified category and the mean number in the reference category; Ratio = 1 means equal means in both categories.

**Table 16:** To be read with Figure 2 (Extended Canter). Summary statistics for total duration (milliseconds) of fixation in Extended Canter and estimated ratio ^2^ between means with 95% confidence interval of the final model ^1^ for interaction between Judging Experience (JE) and Area of Interest (AOI) and Horse-Rider Combination (HRC).

Total Duration of Fixations				RATIO		
	Average	SD	Median	Ave|Ratio	2.50%	97.50%
(Mean in Ref)				2452.3	1546.5	3888.6
Area of Interest						
Back	2279.7	1201.2	2253.5	Ref		
Front	2592.5	1312.0	2464.0	1.1	0.7	1.9
Feet	280.6	470.2	30.0	0.1	0.1	0.2
Rider	1610.2	1029.7	1578.5	0.6	0.4	1.1
Advanced compared to Foundational (Ref)						
Back in Advanced	1876.5	1733.5	1550.0	0.6	0.3	1.2
Front in Advanced	2267.1	1481.7	2550.5	0.7	0.4	1.4
Feet in Advanced	1240.7	1887.8	355.0	2.5	1.3	4.9
Rider in Advanced	842.7	1017.0	371.0	0.4	0.2	0.7
Horse-Rider Combination						
HRC 1	1916.0	1637.4	1742.0	Ref		
HRC 2	1345.0	1269.5	1008.0	0.7	0.5	0.9

Ref = reference category. ^1^ Fixed effects in the full model: Judging Experience (JE) + Area of Interest (AOI) + Horse-Rider Combination (HRC) + Interaction (JE:AOI). ^2^ Ratio between the mean number in the specified category and the mean number in the reference category; Ratio = 1 means equal means in both categories.

**Table 17:** To be read with Figure 3 (Extended Canter). Summary statistics for average duration (milliseconds) of fixation in Extended Canter with estimated difference between means and a 95% confidence interval of the final model ^1^ per Judging Experience (JE) and Area of Interest (AOI).

Average Duration of Fixation (Duration/(Number + 1))						
	Average	SD	Median	Ave|Dif	2.50%	97.50%
(Mean in Ref)				228.4	189.9	266.9
Area of Interest						
Back	228.4	99.6	204.6	Ref		
Front	167.1	61.4	162.6	−61.3	−106.0	−16.6
Feet	57.2	75.4	15.0	−171.2	−215.9	−126.6
Rider	155.8	71.7	162.3	−72.6	−117.3	−27.9
Advanced compared to Foundational (Ref)						
Back in Advanced	164.7	97.6	160.2	−63.7	−121.2	−6.3
Front in Advanced	158.3	93.1	151.4	−8.8	−66.3	48.6
Feet in Advanced	114.5	103.8	92.2	57.4	−0.1	114.8
Rider in Advanced	99.2	78.7	93.8	−56.6	−114.1	0.8

Ref = reference category. ^1^ Fixed effects in the full model: Judging Experience (JE) + Area of Interest (AOI) + Horse-Rider Combination (HRC) + Interaction (JE:AOI).

**Table 18:** To be read with Figure 4 (Extended Canter). Summary statistics for the number of fixations in Extended Canter and estimated ratio ^2^ between means with a 95% confidence interval of the final model ^1^ for Interaction between Judging Experience (JE) and Area of Interest (AOI) and Horse-Rider Combination (HRC).

Number of Fixations				RATIO		
	Average	SD	Median	Ave|Ratio	2.50%	97.50%
(Mean in Ref)				10.7	7.6	15.0
Area of Interest						
Back	8.9	3.6	9.0			
Front	14.2	5.7	14.0	1.7	1.1	2.4
Feet	1.6	2.4	1.0	0.2	0.1	0.3
Rider	8.9	6.4	7.5	1.0	0.7	1.5
Advanced compared to Foundational (Ref)						
Back in Advanced	8.8	5.8	7.5	1.0	0.6	1.7
Front in Advanced	12.2	7.2	11.5	0.8	0.5	1.3
Feet in Advanced	5.8	7.3	2.5	3.4	1.9	6.1
Rider in Advanced	5.2	6.0	2.0	0.6	0.3	0.9
Horse-Rider Combination						
HRC 1	9.9	7.1	9.5			
HRC 2	6.6	6.0	5.5	0.6	0.5	0.8

Ref = reference category. ^1^ Fixed effects in the full model: Judging Experience (JE) + Area of Interest (AOI) + Horse-Rider Combination (HRC) + Interaction (JE:AOI). ^2^ Ratio between the mean number in the specified category and the mean number in the reference category; Ratio = 1 means equal means in both categories.

## Data Availability

The original data presented in the study are openly available in DANS Data Station Life Sciences at https://doi.org/10.17026/LS/9KGVWL.
